# Time Cost of Standardized Nursing Screens in the Emergency Department

**DOI:** 10.5811/westjem.2019.9.44084

**Published:** 2019-10-16

**Authors:** Victoria L. Migdal, Kaitlin Harper, Nazish Haqqani, Bruce Janiak

**Affiliations:** Medical College of Georgia, Augusta University, Department of Emergency Medicine, Augusta, Georgia

## Abstract

**Introduction:**

Various policies require that screening questions be asked of all patients who present to the emergency department (ED). No studies have previously examined the potential time costs of standardized screens. Our objective was to analyze the time nursing spent conducting standardized nursing screens and calculate the corresponding time cost.

**Methods:**

This was a prospective observational study of ED registered nurses (RN) performing triage assessments on adults presenting to the ED. A study author timed nurses while the RN asked five pre-selected questions from their current triage protocol. The time cost of each question was determined by multiplying the length of time spent asking the question each year by the mean hourly wage of RNs at the study hospital. (T/3,600) × V × S; T = mean time per question (in seconds); V = annual patient volume; S = mean hourly RN wage.

**Results:**

We observed 200 triage assessments. During the triage assessments, 130 patients (65%) were asked about pneumococcal vaccine status; 161 (80.5%) about tetanus vaccine status; 184 (92%) about medication allergies; 172 (86%) about influenza vaccine; and 73 (36.5%) about recent travel. The mean time spent per question ranged from 4.37–6.26 seconds. The estimated annual time used to ask the five questions in the study ED is 590.73 hours, which equates to $20,675.50 in nursing costs per year.

**Conclusion:**

There are potential monetary and time costs of standardized screening questions in the ED. The values heavily impact time and cost efficiency in the ED and could be redirected to more pertinent patient care. The required screening questions often have an unclear utility on the care that the patient receives in the ED. Further studies are needed to determine cost effectiveness of required ED screenings.

## INTRODUCTION

The Joint Commission, other medical governing agencies, and various hospital policies mandate that certain screening questions be asked of all patients who come through the emergency department (ED) for evaluation. Before a patient has even seen a physician, they have likely been asked dozens of screening questions as part of the triage or nursing assessment. Screening questions are often implemented with good intentions and some questions serve as public health screening where the ED acts as a safety net.^1^–^3^

The downstream consequences of adding on numerous questions to the ED stay are often not considered. There is the potential for a significant amount of nursing time to be used administering assessments. Additionally, the purpose of triage is to identify and prioritize patients who require immediate treatment over those who do not. The required screening questions often have an unclear benefit on determining triage acuity and on the care that the patient receives in the ED. In many instances the addition of screening questions is based on rudimentary studies that do not examine clinical outcomes or costs.^4^

Screening questions can add time to the triage process and ED wait time, and take nurses away from performing more direct patient care. While any individual question may not take long to ask, when you multiply it by the tens of thousands of patients who pass through the ED and the expanding number of screening questions, it quickly adds up to a significant amount of time. Our objective was to analyze the time nursing spent conducting standardized nursing screens and calculate the corresponding time cost.

## METHODS

This was a prospective observational study of ED RNs performing triage assessments on adults presenting to the ED for medical care in a single academic hospital in the United States. Institutional review board (IRB) approval for this study was obtained from the Augusta University IRB Office. Augusta University Medical Center (AUMC) is an academic, urban hospital with an ED with 83,860 visits during fiscal year (FY) 2018. The mean RN salary at AUMC during FY18 was $35 per hour ($35/hr); this represents the mean for all RNs in the hospital, including ED nurses.

The triage process was observed for all adult patients (age ≥ 18 years) presenting to the ED for treatment. To be included, patients had to go through triage (ie, not directly brought back to room by emergency medical services or to a critical room). Patients were excluded if they were discussing sensitive information (human immunodeficiency virus status, psychiatric complaint) or if they were unable to answer triage and nurse screening questions. Patients and triage nurses were provided an information sheet explaining that the study authors were conducting an observation study of nursing procedures. Verbal consent was obtained from nurses and patients. Patients and nurses were given the opportunity to opt out of being observed. All data collected was anonymous and no personal information was collected.

After reviewing the triage and in-room nurse screening questions asked at AUMC, we selected five questions to be timed.

1. Have you received a pneumococcal vaccine?
2. Have you had a tetanus shot within the last five years?
3. What are your allergies?
4. Have you received a flu shot this year?
5. Any recent travel?

These five questions were selected because they did not impact the patients’ acuity level and all five questions were included in the triage questionnaire. Additional triage questions, such as medical history and history of present illness, were not included in the study because of their potential to impact acuity level.

From July 2018 – January 2019, a total of 200 triage assessments were observed. The study authors would select times throughout the day to observe the triage process and collect data. During the triage assessment, the study authors observed triage nurses as they asked the five pre-selected questions. The nurses were not pre-selected and data was collected on whichever nurse was assigned to work in triage. Not all questions were asked of every triaged patient. The questions asked were at the nurses’ discretion and the data collectors did not interfere with the triage process.

Time was calculated using a stop clock timer. The timer was started as soon as the nurse began asking the question and stopped when the patient completely answered the question and the topic was changed. We calculated the time cost for each question by multiplying the time spent addressing the question each year by the mean hourly wage of AUMC ED RNs. (T/3,600) × V × S, where T = mean time per question (in seconds), V = annual patient volume at AUMC, S = mean hourly RN wage. We used Google Sheets (Google LLC, Mountain View, CA) for all calculations and statistical analysis.

## RESULTS

In total, we observed 200 triage assessments during the study period. During the triage assessments, 130 patients (65%) were asked about pneumococcal vaccine status, 161 (80.5%) about tetanus vaccine status, 184 (92%) about medication allergies, 172 about influenza vaccine (86%), and 73 (36.5%) about recent travel. The mean time spent per question ranged from 4.37–6.26 seconds (Table 1). The estimated annual time used to ask the five questions in the AUMC ED was 590.73 hrs. At a salary of $35/hr, this equates to $20,675.50 in nursing costs per year.

## DISCUSSION

This is a cursory look at the potential monetary and time costs of standardized screening questions in the ED. The calculated values directly affect time and cost efficiency in the ED process and could potentially be redirected to more direct patient care. For just the five observed triage questions alone, we estimated the nursing time cost to our institution to be $20,675.50. This time cost would be significantly increased if we examined additional triage and nurse screening questions. Furthermore, this is just the time spent in a single ED. If all 136.9 million adult ED visits in the U.S. included the five studied questions the screening would take 964,354 hours to complete.^5^ This equates to $33.8 million in nursing costs annually.

The required screening questions are often unrelated to the patient’s chief complaint and have a debatable impact on the medical management in the ED. Questions that may impact care, such as medication allergies, are typically asked by multiple medical providers during the ED visit, and redundancy leads to additional wasted time and cost. It is unclear whether the standardized questions are suitable for triage where the goal is to identify and prioritize patients who require immediate treatment over those who do not. Previous work has shown that triage assessments can have poor inter-rater and intra-rater agreement.^6^ Additional research could evaluate whether the additional screening questions distract the triage nurse from his or her primary goal of assessing acuity and contribute to inconsistency in triage assessments.

If nurses were liberated from the mandated questions, they could potentially have more time for one-on-one patient care and other aspects of patient care, such as medication administration and lab draws. Although we suspect that reducing the number of required questions would free nurses to spend more time on direct patient care and improve efficiency of ED throughput, additional research will be required to study this hypothesis.

Studies evaluating ED screening questions often praise their ability to detect at-risk groups without looking at patient-oriented outcomes or cost. Cost-benefit analyses should be considered prior to mandating additional nurse screening questions as even a few seconds spent on a question adds up to a significant amount of time. A better research agenda is needed to assess the impact of triage questions on patient care.^7^ There is significant potential for future research related to this topic. Further studies are needed to determine cost effectiveness of required ED screenings, including questions included as public health screens. Other potential timesaving measures, such as self-completed triage questionnaires on kiosks, could be researched as well.

## LIMITATIONS

Because this was a prospective observational study, we were unable to definitively state what the time saving would be if the five questions were eliminated. Future projects could implement a treatment group or trial period to evaluate the actual time saving and cost reduction that would occur with questions in the standardized nursing screens. Further, as this was a preliminary observational study, we had a limited sample size of only 200 assessments. Future research would benefit from a larger sample size to obtain more accurate time measurements. Additionally, we did not document the exact number of patients excluded.

Given the non-blinded nature of the study, the Hawthorne effect could have influenced our findings. It is possible that the nurses involved in the study may have subconsciously altered their triage process while being observed. Since they knew they were being observed, they may have been trying to be more efficient and get through their questions faster or conversely they could have been more thorough in their assessment and took longer than when they are not observed. Finally, this was just a limited look at the time spent asking five pre-selected triage questions. Future work needs to be done to analyze the time spent asking additional screening questions, such as fall risk, suicidality, domestic abuse/“safe at home,” and alcohol abuse risk.

## CONCLUSION

Significant ED nursing time is spent asking triage and nurse screening questions. The evidence is unclear as to whether screening questions improve the care that patients receive in the ED. Our data suggest that there is a significant time cost for asking standardized questions, and further cost-benefit analysis must be conducted to determine the usefulness of including these standardized questions as a part of the ED visit.

## Figures and Tables

**Table 1:** Time to obtain answers to give preselected nursing triage questions and monetary cost to the emergency department.

	Mean Time/Question(s) ± SD	Hours/Year^1^	Annual Nursing Cost (Dollars)^2^
Pneumococcal^a^	4.37±1.39	101.68	3,558.97
Tetanus^b^	4.61±1.42	107.33	3,756.68
Allergies^c^	6.26±2.83	145.76	5,101.53
Influenza^d^	4.57±1.42	106.47	3,726.44
Travel^e^	5.56±2.71	129.48	4,531.88
Total	25.36	590.73	20,675.50

1 Based on 83,860 ED patient visits in FY18;

2 Based on mean AUMC RN salary of $35/hr;

a Pneumococcal vaccination status, n=130;

b Tetanus vaccination status, n=161;

c Medication allergies, n=184;

d Influenza vaccination status, n=172;

e Recent travel in last 4 weeks, n=73.
